# Understanding perceived stigma and depression symptoms in maintenance hemodialysis patients: a network perspective

**DOI:** 10.3389/fpsyg.2025.1552518

**Published:** 2025-09-01

**Authors:** Yurong Li, Yaoyao Yang, Shiman Liang, Guanghui Cao, Jinjin Yang

**Affiliations:** ^**1**^Department of Urology, Henan Provincial Key Medicine Laboratory of Nursing, Henan Provincial People’s Hospital: Zhengzhou University People’s Hospital, Henan University People’s Hospital, Zhengzhou, Henan, China; ^2^School of Nursing and Rehabilitation, Cheeloo College of Medicine, Shandong University, Jinan, Shandong, China; ^3^HangZhou Medical College, Lin’an, Zhejiang, China

**Keywords:** perceived stigma, depressive symptoms, maintenance hemodialysis, network analysis, core symptoms, bridge symptoms

## Abstract

**Objectives:**

Patients undergoing maintenance hemodialysis (MHD) experience stigma due to their reliance on machines and changes in appearance, contributing to negative psychological outcomes. Depression symptoms can be considered a product of perceived stigma. However, the inter-relationships between perceived stigma and depression symptoms have not been examined using network analysis among MHD patients. The aim of this study was to model the perceived stigma and depression symptoms network structure, identify its core symptoms, analyze the internal connections between perceived stigma and depression symptoms, as well as identify bridge symptoms in the stigma-depression network.

**Methods:**

This study included 301 MHD patients in a cross-sectional design. The participants completed self-reported measures of perceived stigma and depressive symptoms. A cross-sectional network analysis was performed using the R language to model the network structure and identify core and bridge symptoms in the network.

**Results:**

The core symptoms of perceived stigma from the network analysis were SIS5 “Feel others avoid me because of my illness” (Strength = 1.258, Betweenness = 32, Closeness = 0.00303), SIS13 “Feel others think I am to blame” (Strength = 1.142, Betweenness = 62, Closeness = 0.00298), and SIS11 “My job security has been affected” (Strength = 1.108, Betweenness = 72, Closeness = 0.00313). The core symptoms of depression were PHQ6 “Worthlessness” (Strength = 1.213, Betweenness = 13, Closeness = 0.00211), PHQ1 “Anhedonia” (Strength = 1.048, Betweenness = 20, Closeness = 0.0150), and PHQ2 “Sad mood” (Strength = 1.012, Betweenness = 8, Closeness = 0.0164). Regarding the combination network, results showed that SIS2 “Some people think I am less competent” (Bridge Strength = 0.917) and SIS11 “My job security has been affected” (Bridge Strength = 0.783) were the two most prominent bridge nodes.

**Conclusion:**

This research reveals the core and bridge symptoms in different symptomatic profiles (such as perceived stigma, depression symptoms, and their combination networks), which can be targeted for treatment personalization and aid in diminishing depressive symptoms and perceived stigma among MHD patients.

## Introduction

End-stage renal disease (ESRD) has gradually evolved into a global public health problem that seriously endangers human health (Ghimire et al., 2021). By 2030, the number of people with ESRD worldwide is expected to reach 5.439 million (Shirai et al., 2022). Patients with ESRD need renal replacement therapy such as dialysis or transplantation to survive (Webster et al., 2017), with maintenance hemodialysis (MHD) being the most widely used method (Htay et al., 2021). Data from the Chinese National Renal Data System (CNRDS) showed that the total number of MHD patients in China had reached 916,647 by the end of 2023 (Chen, 2024).

Despite MHD prolonging survival, this treatment is not curative (Lu et al., 2022). Physical appearance changes related to the disease and long-term dialysis (e.g., skin hyperpigmentation, urea frost, desquamation, or fistulas in the arms), diminished status, reduced employment opportunities, combined with public misconceptions and prejudice towards uremia, can lead to stigmatization (Kerklaan et al., 2020). Link and Phelan (2006) conceptualize stigma as a dynamic, stepwise process comprising five interrelated components: labeling, stereotyping, separation, status loss, and discrimination, which can be categorized by source into three distinct types: actual stigma, perceived stigma, and internalized stigma (Link and Phelan, 2001). Perceived stigma refers to an individual’s awareness or perception of the negative stereotypes that the general public may hold due to changes in their physical, psychological, and social status as a result of illness (Stynes et al., 2022). Previous studies have indicated that perceived stigma is both prevalent and severe among MHD patients (Casey et al., 2014; Chen et al., 2021a). This stigma may be further exacerbated within the context of China’s collectivist culture, which places a strong emphasis on face-saving (Fessler, 2004). Remarkably, scholars have found that rather than being a singular symptom (Jing et al., 2024), perceived stigma may be deconstructed into a network of more specific concerns, which are complexly interrelated (Wei et al., 2020). Prior studies have examined the various dimensions and overall scores of perceived stigma in MHD patients (He et al., 2022; Lu et al., 2022), but the internal structure within perceived stigma remains elusive.

Perceived stigma exacerbates illness-related stress and triggers maladaptive coping strategies, including the concealment of the disease and social withdrawal. These behaviors contribute to non-adherence to treatment and delays in seeking help, thereby undermining therapeutic outcomes and diminishing quality of life (Hatzenbuehler et al., 2013). Beyond that, it has been shown that long-term exposure to stigma causes individuals to internalize negative self-concepts, thereby leading to self-deprecation and social isolation, which are contributing factors to poorer psychological outcomes (Fife and Wright, 2000). As one of the prevalent psychological consequences, depressive symptoms are common in MHD patients, with reports indicating that 50% of them experience such symptoms (Thomas et al., 2017). These depressive symptoms can further impair medical treatment adherence, reduce dialysis efficacy, compromise immune system function, and affect nutritional status, potentially leading to adverse outcomes and a decline in quality of life (Cirillo et al., 2018). Additionally, depressive symptoms are significantly correlated with increased mortality rates among MHD patients (Sy et al., 2019). Previous literature has shown a significant association between stigma and depressive symptoms in people with certain diseases, such as multiple sclerosis (Cadden et al., 2018), cancer (Pham et al., 2021), and inflammatory bowel disease (Gamwell et al., 2018). Another study in this field also showed that perceived stigma was directly and positively correlated with depressive symptoms (Lu et al., 2022).

Although the aforementioned studies have investigated depression, stigma symptoms, and their correlations in MHD patients, most of them were based on traditional latent variable theory and the assumption of local independence. This means that depressive and stigma symptoms were considered as a result of common latent variables, with the total score used to explain symptom severity while disregarding symptom interaction (Chen et al., 2021b). However, during the experience of mental disorders, the interaction between symptoms is a common phenomenon (Fan et al., 2024). In recent years, the network theory of mental disorders (NTMD; Cramer et al., 2010; Borsboom and Cramer, 2013; Borsboom et al., 2018) has received increased attention as an alternative model that may overcome these persistent limitations. According to NTMD, psychological symptoms are not caused by a latent variable but rather by a dynamic network of interactive symptoms, and the development and maintenance of mental diseases result from interactions between various symptoms (Mcnally et al., 2015). The network structure consists of two parts: edges and nodes. Nodes represent psychological symptoms, while edges represent their connections (Cai et al., 2020). Network analysis, a novel statistical approach corresponding to NTMD, can assess the importance of nodes within a broader network framework by evaluating their centrality (Jing et al., 2024). In simpler terms, this approach offers a method to analyze the relationships between symptoms in a network of mental disorders and identify the most crucial symptoms based on their strong connections with other symptoms (Wei et al., 2020). By directly targeting core symptoms, therapeutic interventions can more efficiently disrupt maladaptive symptom networks, thereby enabling precise prevention of adverse clinical outcomes (Wang et al., 2023). Moreover, network analysis can identify bridge symptoms that potentially contribute to the development and maintenance of comorbid mental conditions (Jones et al., 2021). This approach facilitates developing tailored interventions that simultaneously target multiple psychological disorders (Wang et al., 2023). Significantly, although advancements in dialysis technology have improved patient life expectancy (Zhang et al., 2022), treatment non-adherence and the decline in quality of life, primarily due to perceived stigma and depression, now represent the main obstacles to long-term survival. This highlights the clinical necessity for the development of precision interventions that target core and bridging symptoms through network analysis, aiming to simultaneously address perceived stigma and depressive symptoms. However, to date, only a limited number of studies have employed network analysis to investigate these phenomena in patients with MHD.

In summary, the current study aims to employ cross-sectional network analysis to: (1) model the network structure of perceived stigma and identify its core symptoms; (2) model the network structure of depression symptoms and identify its core symptoms; and (3) examine the internal connections between perceived stigma and depression symptoms, as well as identify bridge symptoms within the stigma-depression network.

## Methods

### Study design and participants

This study utilized a cross-sectional design and employed convenience sampling to recruit patients with ESRD undergoing MHD between July and November 2020 at three tertiary grade A hospitals located in Jinan, Shandong Province, China. The inclusion criteria were as follows: (1) a diagnosis of end-stage renal disease and at least 3 months of hemodialysis treatment, (2) age above 18 years, and (3) voluntary participation. Patients with cognitive impairment, a history of mental illness, or an inability to communicate or comprehend questionnaires were excluded. Sample size was determined per Memon et al. (2020), requiring ≥165 participants for network analysis (5–6 cases per node; 33 nodes). Accounting for 10% invalid responses, the total sample size was 182. This research project obtained ethical approval from Shandong University’s School of Nursing and Rehabilitation Ethics Committee, and informed consent was obtained from all participating patients prior to their involvement in this study.

### Data collection

Given that the participants were receiving hemodialysis treatment at the time of the survey, filling out the paper questionnaire was not practical, yet they could manage their mobile phones. Therefore, an electronic questionnaire was designed and provided to them. We sent the participants a link to the electronic questionnaire. After providing electronic written informed consent, patients could access the data collection form and questionnaire. Participants were instructed to fill out the questionnaire on their mobile phones during the survey. To avoid missing data, all questions are required to be completed before submission. Additionally, two random attention check items were included throughout the questionnaire, and participants who answered these items incorrectly were excluded from analysis. Participants were informed about their right to withdraw from the study at any time. As a token of appreciation, each participant who completed the survey received a gift. A total of 301 valid responses were obtained from the 341 distributed questionnaires, resulting in a completion rate of 88.27%. The questionnaires were systematically organized and inputted into the database, after which the data entry was verified by an independent researcher to minimize the risk of errors.

### Measures

#### Socio-demographic and clinical information

A questionnaire, developed in-house, was used to gather socio-demographic (age, gender, marital status, financial burden, education level, religious belief, and employment status) and clinical characteristics (primary disease types, dialysis duration, complications count, and renal transplantation experience) of MHD patients.

#### Perceived stigma

The Social Impact Scale (SIS; Fife and Wright, 2000) was utilized to assess perceived stigma. This scale comprises 24 items divided into four dimensions: social rejection (9 items), financial insecurity (3 items), internalized shame (5 items), and social isolation (7 items). Social rejection refers to the experience of individuals perceiving discrimination in both professional and societal contexts, which encompasses a sense of diminished respect from others, perceived incompetence, avoidance by others, and apparent discomfort in their presence. Financial insecurity is a direct result of workplace discrimination, leading to insufficient job stability and income. This discriminatory treatment directly impacts individuals’ self-perception and interactions with others. Internalized shame involves feeling disconnected from people who are in good physical condition, assigning blame to oneself for the illness, and feeling compelled to hide the illness. Social isolation refers to a sense of societal alienation in the conventional sociological context (Fife and Wright, 2000), encompassing emotions of solitude, disparity among individuals, and lack of purpose. The validation of the SIS in previous research has been conducted for its Chinese adaptation (Guan et al., 2011). Participants responded to the items using a 5-point Likert scale ranging from strongly agree (1) to strongly disagree (5). Higher scores indicate higher levels of perceived stigma. The reliability coefficients for the SIS in this study were found to be 0.932 for Cronbach’s alpha (*α*) and 0.933 for McDonald’s omega (*ω*).

#### Depressive symptoms

The nine-item Patient Health Questionnaire (PHQ-9) (Kroenke et al., 2001) was used to measure depressive symptoms. The reliability and validity of the Chinese adaptation of the PHQ-9 have been well-documented in prior studies (Bian et al., 2009). The items were evaluated using a 4-point scale that ranged from 0 (not at all) to 3 (nearly every day). Higher scores indicate more severe depressive symptoms. In the present investigation, the Cronbach’s alpha of the PHQ-9 was 0.915, and McDonald’s omega was also 0.915.

### Data analyses

SPSS 26.0 software was used for descriptive analysis, and the socio-demographic and clinical characteristics of the participants were described by frequency and percentage. R 4.4.1 software was used for network analysis. We performed three main analyses: network estimation, centrality and predictability measures, and accuracy and stability estimations.

### Network estimation

We used the “qgraph” (version 1.9.2) and “bootnet” (version 1.5.0) package in R for network estimation and visualization. The network structure was estimated using the graph least absolute shrinkage and selection operator (gLASSO) method, based on the Extended Bayesian Information Criterion (EBIC; Chen and Chen, 2008). This regularization procedure shrinks all edges and sets edges with small partial correlations to zero to obtain a parsimonious and sparse network which is more stable and easier to interpret (Foygel and Drton, 2010; Foyger Barber and Drton, 2015). Meanwhile, the tuning parameter (hyperparameter *γ*) was set to 0.5 to well balance sensitivity and specificity of picking out true edges (Epskamp et al., 2018). In the visualized networks, each item is represented as a “node,” and the link between two nodes is shown as an “edge.” The thickness of edge corresponds to association strength, where solid lines represent positive partial correlations and dashed lines denote negative partial correlations.

### Centrality and predictability measurements

To identify central items in the network, three primary centrality indices were typically calculated using the “centralityPlot” function in the “qgraph” package (version 1.9.2): “Strength” (the total sum of absolute weights of edges connecting a node to all other nodes), “Betweenness” (the number of shortest paths linking any two nodes), and “Closeness” (the inverse of the sum of lengths of all shortest paths). Moreover, we used the R package networktools (version 1.5.0) to identify bridge items and calculate the bridge strength centrality index connecting the two symptom clusters (e.g., perceived stigma and depression), three bridge indices were commonly computed: “Bridge Strength,” “Bridge Betweenness,” and “Bridge Closeness.” Higher values for these indices indicate greater centrality or bridge centrality. Among them, “Strength” is considered the most crucial index. When the numerical ranking of the three indicators is inconsistent, the ranking result of “Strength” is generally used as the standard (Hallquist et al., 2021; Epskamp et al., 2018). Furthermore, we utilized the R package mgm (versions 1.2–12; Haslbeck and Fried, 2017) to calculate the predictability of each node. Predictability refers to the extent to which a node’s variance can be accounted for by its neighboring nodes, and this index could characterize the controllability of the network (Wei et al., 2020).

### Estimating the accuracy and stability of the network

Following the proposal of Epskamp et al. (2018), network solution robustness was assessed using the R package bootnet (version 1.5.0) to evaluate edge weight accuracy and centrality index stability. The accuracy of network edges was estimated by using bootstrapped 95% confidence intervals (CIs) of the edge weights. The smaller the overlap between 95% CIs, the more accurate the edge estimate. The case-dropping subset bootstrap was executed to calculate the centrality stability coefficient (CS-C) and thereby assess the stability of the centrality index. The CS-C value reflects the maximum proportion of samples that may be excluded while maintaining, with 95% probability, a correlation of at least 0.70 between the original centrality indices. Generally, the CS-C should be no less than 0.25 and ideally above 0.50 (Epskamp et al., 2018).

## Result

### Study sample

A total of 301 patients with MHD were included in the study, comprising 176 males (58.4%) and 125 females (41.5%). The majority of patients fell within the age range of 46–64 years old (48.5%). Among them, 238 were married (79.1%) and 63 were unmarried (20.9%). Additionally, 92 were employed (30.6%) and 209 were non-employed (69.4%). The duration of dialysis varied from 4 months to a maximum of 20.8 years, with an average duration of approximately 4.55 years. Out of all participants, 75 expressed their intention for transplantation (24.9%), while the remaining 226 did not express such intentions. Table 1 showed the socio-demographic and clinical characteristics of the 301 MHD patients.

**Table 1:** Socio-demographic and clinical characteristics of participants (*N* = 301).

Characteristic	*N* (%) or (*M* ± *SD*)
Age	51.98 ± 14.31
Gender	
Male	176(58.5)
Female	125(41.5)
Marital status	
Married	238(79.1)
Single/Divorced/Windowed	63(20.9)
Financial burden	
No	70(23.2)
Low	108(35.9)
Moderate	90(29.9)
High	33(11.0)
Education level	
Primary school or below	24(8.0)
Junior high school	90(29.9)
Senior high school or technical secondary school	109(36.2)
Junior college or bachelor’s degree or above	78(25.9)
Religious belief	
Yes	25(8.3)
No	276(91.7)
Employment status	
Employed	92(30.6)
Unemployed	209(69.4)
Primary disease types	
Chronic glomerulonephritis	39(13.0)
Diabetic nephropathy	90(29.9)
Hypertensive nephropathy	98(32.6)
Other	74(24.6)
Duration of dialysis (year)	4.55 ± 3.76
Number of complications	1.99 ± 1.14
Renal transplantation experience	
Yes	20(6.4)
No	281(93.6)

### Network structure

**Figure 1:**
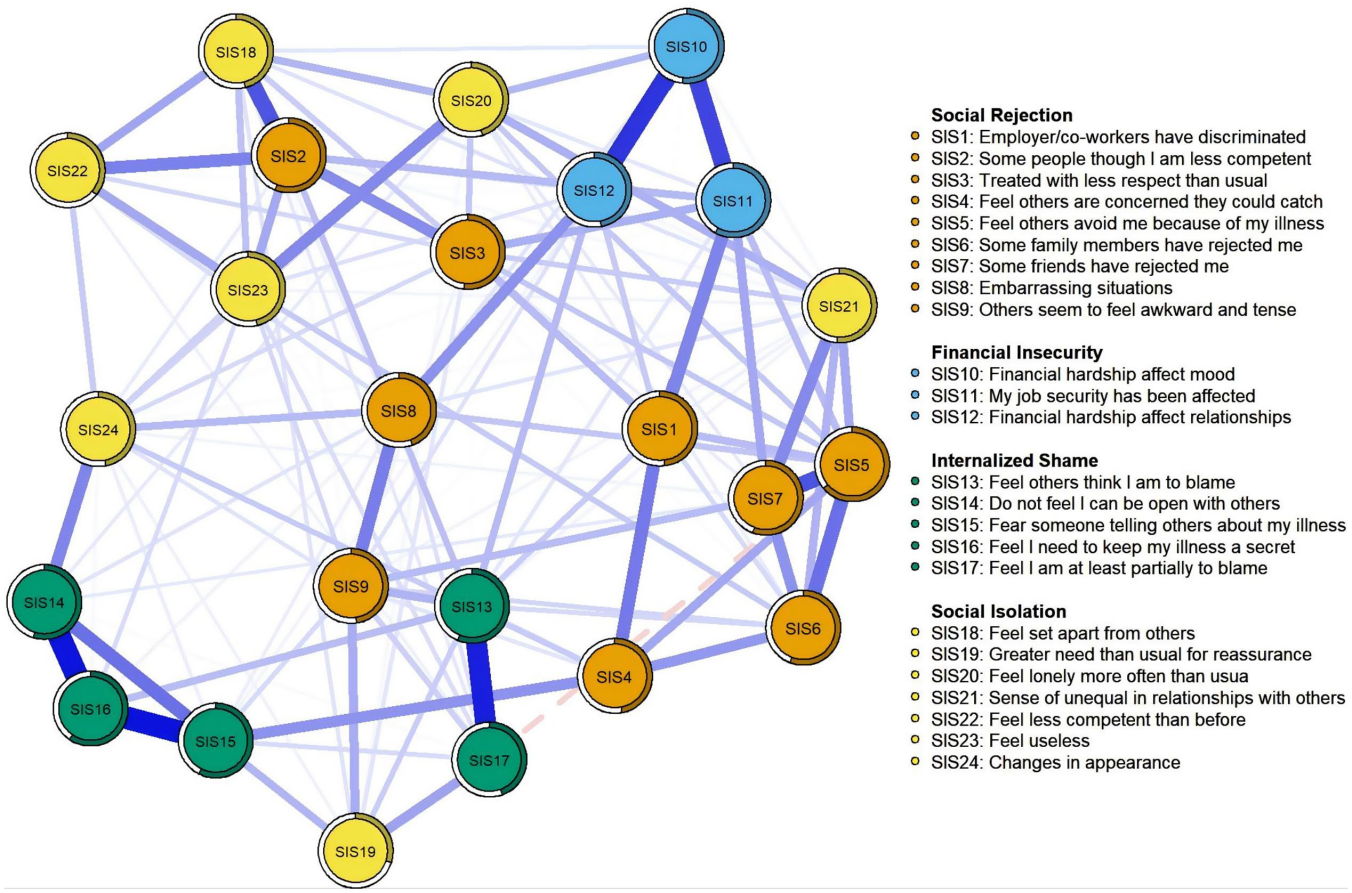
Network analysis visualization for perceived stigma.

The visualization of perceived stigma network was shown in Figure 1. In total, 136 (49.28%) non-zero edges were displayed out of a possible 276 edge. The predictability of all nodes in the network ranges from 29.6 to 64.7%. SIS5 “Feel others avoid me because of my illness” (64.7%) in the network had the highest predictability, followed by SIS11 “My job security has been affected” (58.1%) and SIS16 “Feel I need to keep my illness a secret” (57.8%) (see Table 2). The predictability of each node is shown as a circle around it in Figure 1. Additionally, SIS5 (Strength = 1.258, Betweeness = 32, Closeness = 0.00303), SIS13 (Strength = 1.142, Betweeness = 62, Closeness = 0.00298), and SIS11 (Strength = 1.108, Betweeness = 72, Closeness = 0.00313) were the core symptoms in the network. Other details were provided in Figure 1 and Table 2.

**Figure 2:**
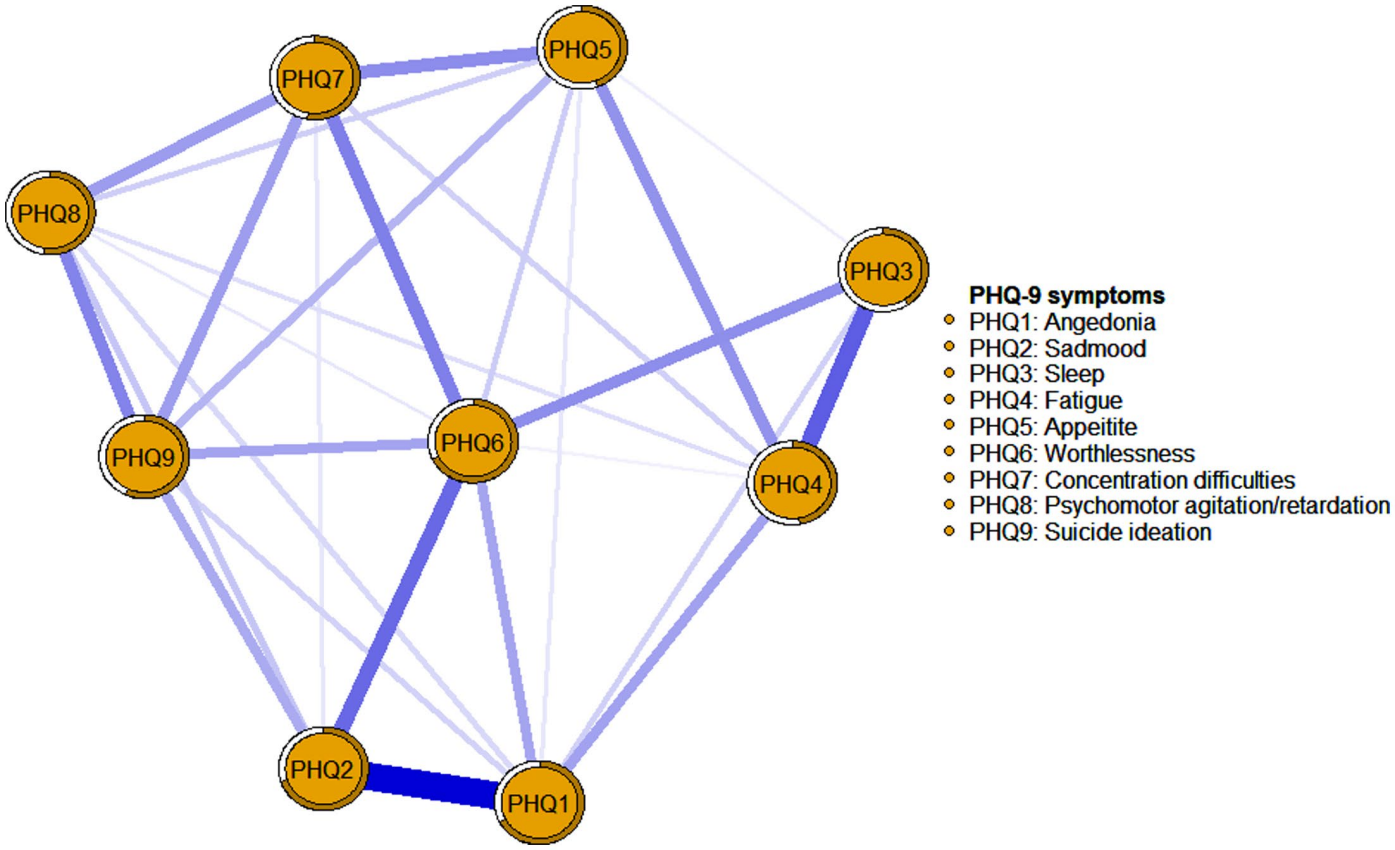
Network analysis visualization for depression symptom.

**Table 2:** Index of node centrality and predictability of perceived stigma.

Symptoms	Network of perceived stigma			
	Strength	Closeness	Betweenness	Predictability
SIS1 Employer/co-workers have discriminated	0.831	0.00296	30	0.477
SIS2 Some people though I am less competent	1.071	0.00282	60	0.562
SIS3 Treated with less respect than usual	0.942	0.00276	6	0.517
SIS4 Feel others are concerned they could “catch”	0.839	0.00290	58	0.488
SIS5 Feel others avoid me because of my illness	1.258	0.00303	32	0.643
SIS6 Some family members have rejected me	0.945	0.00280	14	0.517
SIS7 Some friends have rejected me	0.960	0.00287	30	0.558
SIS8 Embarrassing situations	0.834	0.00271	24	0.443
SIS9 Others seem to feel awkward and tense	0.992	0.00267	30	0.477
SIS10 Financial hardship affect mood	0.809	0.00297	48	0.522
SIS11 My job security has been affected	1.108	0.00313	72	0.581
SIS12 Financial hardship affect relationships	0.886	0.00282	34	0.482
SIS13 Feel others think I am to blame	1.142	0.00298	62	0.566
SIS14 Do not feel I can be open with others	0.890	0.00249	18	0.550
SIS15 Fear someone telling others about my illness	1.055	0.00267	42	0.577
SIS16 Feel I need to keep my illness a secret	0.931	0.00266	22	0.578
SIS17 Feel I am at least partially to blame	0.836	0.00262	8	0.440
SIS18 Feel set apart from others	0.876	0.00239	2	0.460
SIS19 Greater need than usual for reassurance	0.577	0.00233	2	0.300
SIS20 Feel lonely more often than usual	0.903	0.00261	14	0.491
SIS21Sense of unequal in relationships with others	0.912	0.00249	4	0.514
SIS22 Feel less competent than before	0.680	0.00242	8	0.409
SIS23 Feel useless	0.933	0.00268	18	0.465
SIS24 Changes in appearance	0.852	0.00261	26	0.466

The visualization of depression symptoms network was shown in Figure 2. Overall, 28 (77.78%) non-zero edges were displayed out of a possible 36 edges, and most of the edges were positive. The predictability of all nodes in the network ranges from 40.5 to 70.1%. PHQ2 “Sad mood” (70.1%) in the network had the highest predictability, followed by PHQ6 “Worthlessness” (67.7%) and PHQ1 “Anhedonia” (67.2%) (see Table 3). The predictability of each node is shown as a circle around it in Figure 2. PHQ6 “Worthlessness” (Strength = 1.213, Betweeness = 13, Closeness = 0.00211) was the core symptom in the network. All relevant details were presented in Figure 2 and Table 3.

**Table 3:** Index of node centrality and predictability of depression symptom (*n* = 301).

Symptoms	Network of depression symptom			
	Strength	Closeness	Betweenness	Predictability
PHQ1 Anhedonia	1.048	0.0150	2	0.672
PHQ2 Sad mood	1.012	0.0164	8	0.701
PHQ3 Sleep difficulties	0.600	0.0140	2	0.405
PHQ4 Fatigue	0.810	0.0142	6	0.473
PHQ5 Appetite changes	0.770	0.0141	6	0.442
PHQ6 Worthlessness	1.167	0.0187	14	0.677
PHQ7 Concentration difficulties	0.896	0.0169	12	0.529
PHQ8 Psychomoto agitation/retardation	0.748	0.0118	0	0.523
PHQ9 Suicide ideation	0.910	0.0156	0	0.567

**Figure 3:**
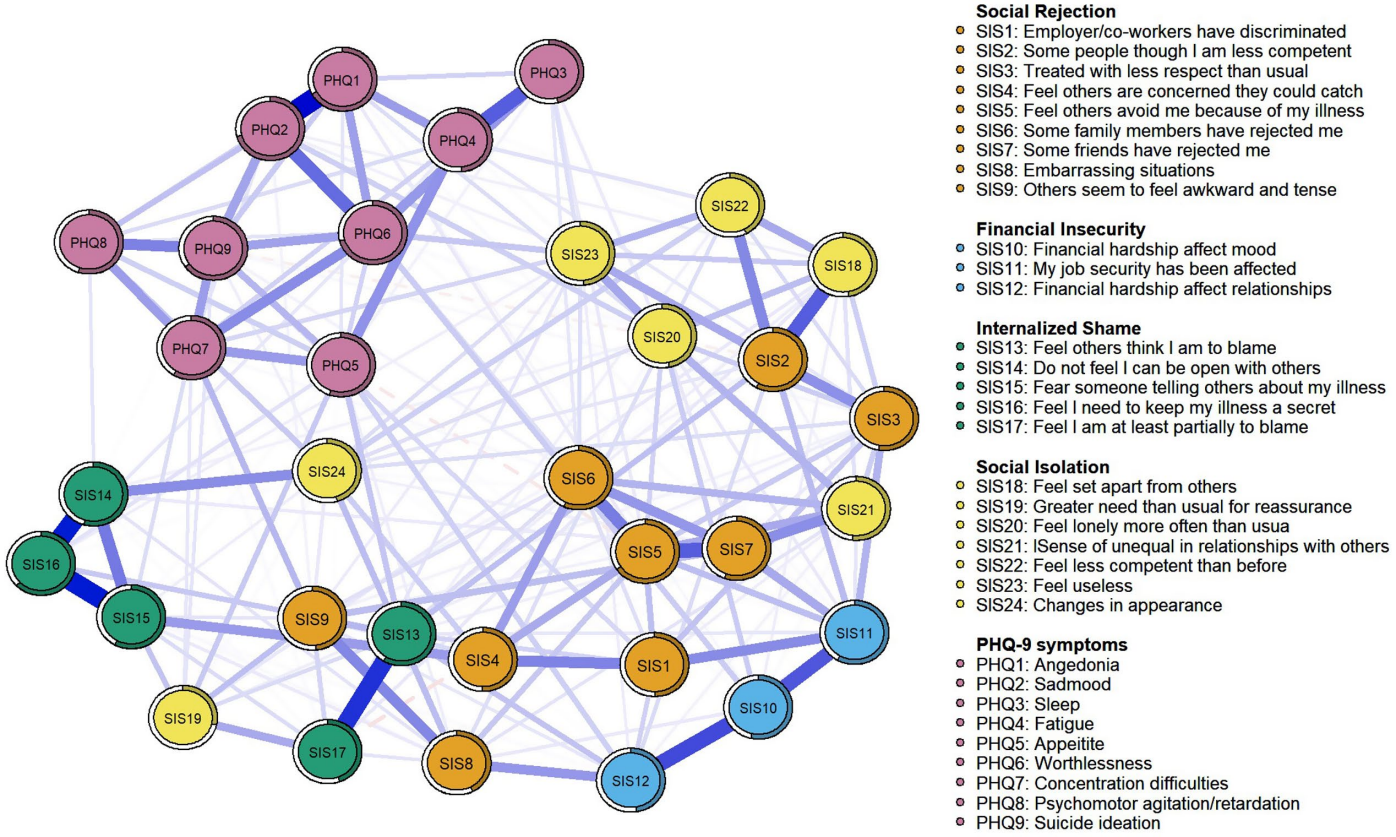
Network analysis visualization for the combination network.

**Figure 4:**
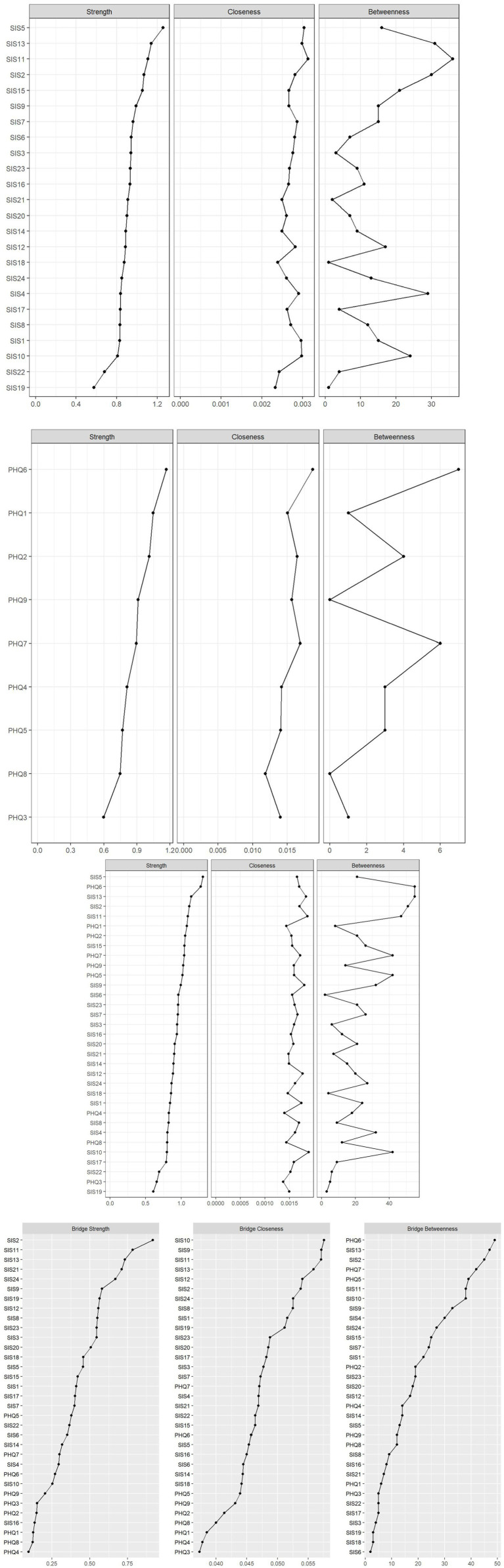
Centrality metrics of perceived stigma network (above) and centrality metrics of depression symptoms network(below). Centrality (above) and bridge centrality (below) metrics of the combination network.

To assess the relationship between perceived stigma and depression symptom, we developed a combined partial correlation network. The visualization of the network was presented in Figure 3. In total, the combination network displayed 210 (39.77%) non-zero edges out of a possible 528 edges. The predictability of all nodes ranges from 28.6 to 71.2%. PHQ6 “Worthlessness” (71.2%) in the combination network had the highest predictability, followed by PHQ2 “Sad mood” (70.3%) and PHQ1 “Anhedonia” (70.0%) (see Table 4). The predictability of each node was shown as a circle around it in Figure 3. Additionally, in terms of nodes centrality in the combination network model, SIS5 “Feel others avoid me because of my illness” (Strength = 1.305) had the highest strength, followed by PHQ6 “Worthlessness” (Strength = 1.273), SIS13 “Feel others think I am to blame” (Strength = 1.141), and SIS2 “Some people though I am less competent” (Strength = 1.113) all of which emerged as core symptoms in understanding the perceived stigma and depression symptom network in MHD patients. According to the bridge strength shown in Figure 4, the results identified SIS2 “Some people though I am less competent” (Bridge Strength = 0.917) and SIS11 “My job security has been affected” (Bridge Strength = 0.783) as the two most prominent bridge variables. All relevant details were presented in Figure 3 and Table 4.

**Figure 5:**
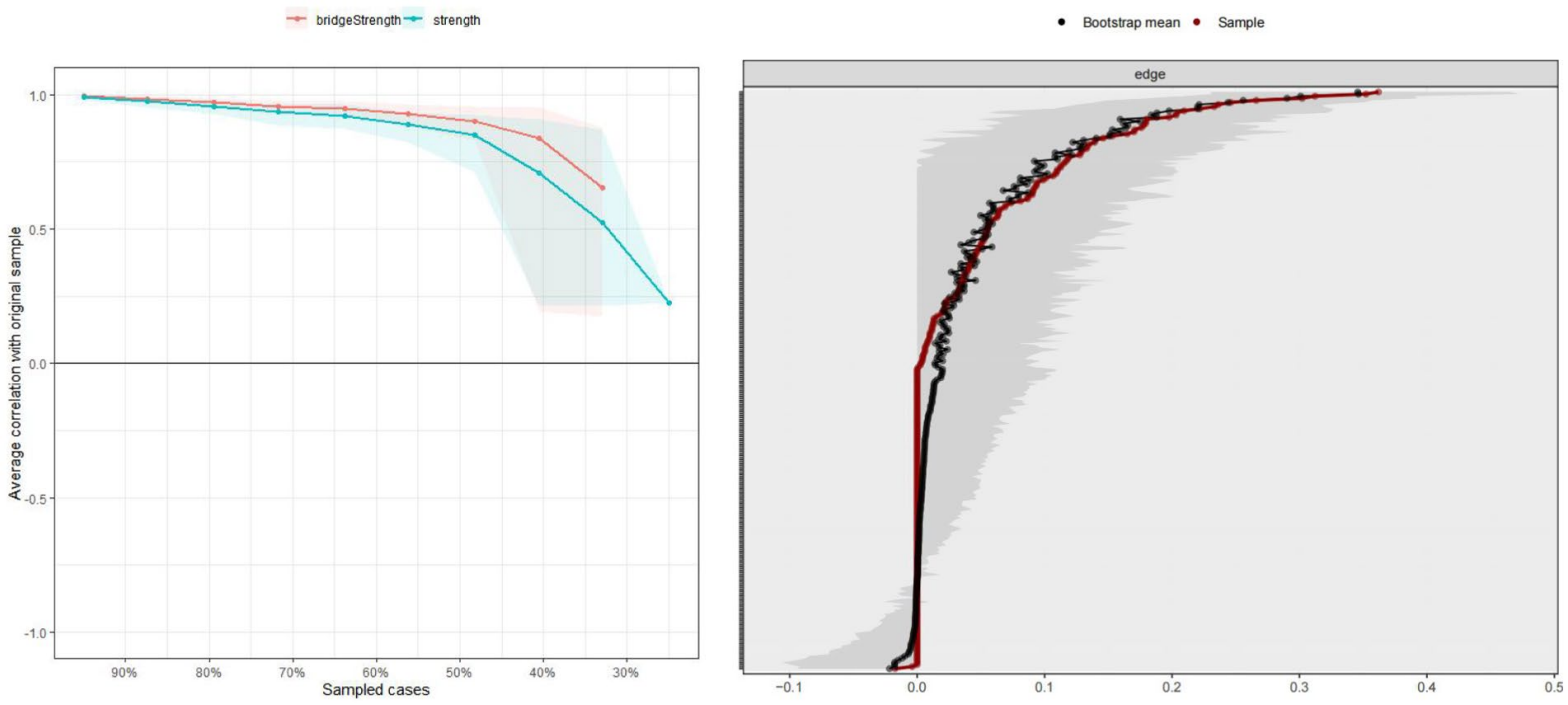
Accuracy and stability of the combination network.

**Table 4:** Index of node centrality, bridge centrality and predictability of combination.

Symptoms	Strength	Bridge Strength	Predictability
SIS1 Employer/co-workers have discriminated	0.844	0.410	0.494
SIS2 Some people though I am less competent	1.113	0.917	0.577
SIS3 Treated with less respect than usual	0.944	0.546	0.529
SIS4 Feel others are concerned they could “catch”	0.808	0.294	0.509
SIS5 Feel others avoid me because of my illness	1.305	0.455	0.654
SIS6 Some family members have rejected me	0.961	0.351	0.572
SIS7 Some friends have rejected me	0.954	0.399	0.558
SIS8 Embarrassing situations	0.823	0.551	0.420
SIS9 Others seem to feel awkward and tense	0.993	0.580	0.483
SIS10 Financial hardship affect mood	0.798	0.253	0.538
SIS11 My job security has been affected	1.092	0.783	0.584
SIS12 Financial hardship affect relationships	0.885	0.557	0.476
SIS13 Feel others think I am to blame	1.141	0.732	0.589
SIS14 Do not feel I can be open with others	0.897	0.317	0.548
SIS15 Fear someone telling others about my illness	1.044	0.419	0.580
SIS16 Feel I need to keep my illness a secret	0.939	0.136	0.617
SIS17 Feel I am at least partially to blame	0.788	0.401	0.451
SIS18 Feel set apart from others	0.857	0.457	0.467
SIS19 Greater need than usual for reassurance	0.608	0.564	0.286
SIS20 Feel lonely more often than usual	0.908	0.507	0.467
SIS21Sense of unequal in relationships with others	0.903	0.711	0.523
SIS22 Feel less competent than before	0.689	0.365	0.442
SIS23 Feel useless	0.955	0.546	0.504
SIS24 Changes in appearance	0.864	0.669	0.497
PHQ1 Anhedonia	1.079	0.125	0.700
PHQ2 Sad mood	1.055	0.148	0.703
PHQ3 Sleep difficulties	0.656	0.152	0.460
PHQ4 Fatigue	0.826	0.095	0.484
PHQ5 Appetite changes	1.017	0.378	0.554
PHQ6 Worthlessness	1.273	0.269	0.712
PHQ7 Concentration difficulties	1.042	0.299	0.589
PHQ8 Psychomotor agitation/retardation	0.802	0.122	0.562
PHQ9 Suicide ideation	1.029	0.205	0.611

We evaluated the stability of the combination network by estimating the CS-C value. The case-dropping bootstrap procedure for the node strength and bridge strength was shown in Figure 5. The CS-C for node strength and bridge strength were 0.503 and 0.518, respectively, indicating that the network model was adequately stable. Furthermore, the results of the 95% confidence interval for the edge weights showed a small bootstrapped confidence interval around the most estimated edge weights, resulting in high accuracy (Figure 5).

## Discussion

This study was, to our knowledge, the first to use network analysis to graphically present perceived stigma, depression symptoms, and the stigma-depression network in a general MHD sample. Although the results were based on cross-sectional data, both network edges and the centrality metrics were stable, which increased the reliability of drawing conclusions.

We found that the item within the dimension of social rejection, specifically “Feel others avoid me because of my illness” (SIS5) exhibits the highest strength in the perceived stigma network, indicating its significance for the onset or persistence of stigma experiences among MHD patients. This aligns with past evidence, which found that “Perceptions of others’ avoidance due to one’s illness” was the most prominent central node in the stigma network in patients with depression and breast cancer (Cai et al., 2022; Li et al., 2024). This item reflects the social alienation due to disease-related changes. According to its definition, stigma draws attention to specific attributes that differentiate certain groups from the rest of society (Latalova et al., 2014). For ESRD, the lifelong dependence on machines for survival and the changes in physical appearance associated with long-term dialysis (e.g., fistulas in the arms or skin hyperpigmentation) set MHD patients apart from others in life scenarios and workplaces, all of which lead to feelings of “otherness” and isolation (Cai et al., 2022). This feeling can trigger maladaptive coping mechanisms such as disease concealment and social withdrawal, which drive treatment non-adherence and delayed help-seeking, ultimately compromising both therapeutic outcomes and quality of life (Hatzenbuehler et al., 2013). These consequences may further heighten perceived stigma (Jacoby et al., 2005). Besides SIS5, we also found that “Feel others think I am to blame” (SIS13), an item within the dimension of internalized shame, was the second strongest node in the perceived stigma network. This finding is consistent with the conclusion of Perlick (2001) that internalization of stigma has a more profound adverse effect on patients than tangible experiences of social exclusion and discrimination. For MHD patients, the perception that others blame them for their condition often stems from societal misconceptions about the causes of ESRD. These misconceptions may include stereotypes about unhealthy lifestyles, genetic predisposition, or even moral failures. When patients internalize these societal labels, they may begin to experience a cycle of self-blame and negative self-worth. This cycle exacerbates depression, anxiety, and other psychological symptoms (Link et al., 1997; Link, 1982). Meanwhile, the social withdrawal that accompany internalized stigma further isolate the patients, exacerbating their feelings of stigma.

“My job security has been affected” (SIS11), which reflects a specific consequence of discrimination in the workplace (Fife and Wright, 2000), was the third strongest node in the network. Similarly, as pointed out by Ryan et al. (1980), perceived stigma is highly dependent on whether an individual has experienced workplace discrimination. Due to the strict dialysis schedule, patients must frequently miss work, and symptoms like fatigue and difficulty concentrating can also impair performance in jobs requiring high concentration or physical labor, thereby reducing job stability. Moreover, some employers may lack sufficient understanding of the health conditions and treatment requirements of patients, which may lead to biases and misconceptions, causing patients to face unfair treatment during job seeking and employment, further compromising their job stability. Studies in ESRD patients have shown that sustained employment is often associated with individual self-esteem, self-efficacy, and the maintenance of social relationships (Kutner and Zhang, 2017). When illness threatens job security, patients may feel their self-worth is questioned, leading to intense feelings of stigma. Another potential explanation is that unemployment results in the loss of workplace social connections and status, meaning the deprivation of social resources or increased social isolation (Zhao et al., 2021). This, in turn, contributes to the formation of stigma.

In the current study, “Worthlessness” (PHQ6) was found to have the highest strength value in the depression symptoms network. This is partially concordant with previous network analyses in patients after pacemaker implantation, which found that PHQ6 is one of the central symptoms among depression symptoms (Lin et al., 2023). Similar to patients with pacemakers, participants with ESRD are required to frequently return to the hospital and rely on their families for long-term MHD treatment, resulting in disruptions to their daily routines and an inability to fulfill familial and job responsibilities, hence they may experience increased feelings of worthlessness. At the same time, prolonged dialysis treatment may increase personal and financial burdens for both their families and the healthcare system, thereby contributing to a heightened sense of worthlessness in affected patients. Furthermore, worthlessness, as one of the cognitive triad of depression (e.g., helplessness, hopelessness, and worthlessness), is more likely to play a key role in the occurrence and development of depressive symptoms (Garima, 2020). This study also found that “Sad mood” (PHQ2) and “Anhedonia” (PHQ1) were the second and third strongest nodes in the network. The findings are consistent with the diagnostic criteria for depression as outlined in the Diagnostic and Statistical Manual of Mental Disorders (DSM-5), where “sad mood” and “loss of interest or pleasure” are core symptoms for the diagnosis of depression. Additionally, “sad mood” is also identified as the most significant negative emotion in maintaining depression (Yang et al., 2022a; Yang et al., 2022b). In brief, addressing core symptoms like “worthlessness,” “sad mood” and “anhedonia” is crucial and could be a focus for future interventions, as these symptoms have the greatest potential to impact the overall manifestation of depressive symptoms. Previous research has also discovered a correlation between patients who reported a higher prevalence of core symptoms of depression initially and their likelihood of developing major depressive disorder later on, in contrast to those who reported more peripheral symptoms (Boschloo et al., 2016). These findings suggest that future research should place greater emphasis on assessing the performance of worthlessness, sadness, and anhedonia in MHD patients, while also guiding patients toward experiencing positive emotions and fostering a positive outlook during health education and nursing interventions. Additionally, timely psychological counseling should be provided to help patients develop a constructive self-perception, acknowledge their self-worth, and encourage them to express and release their emotions, ultimately leading to a broader reduction in overall levels of depression.

In the combined network, we discovered that perceived stigma and depression symptoms are distinct yet related constructs in terms of their relationship. These two subnetworks remained essentially unchanged after the combination, and the core symptoms in both the perceived discrimination symptom network (“Feel others avoid me because of my illness”) and the depressive symptom network (PHQ6 “Worthlessness”) were also identified as core symptoms in the combined network. More importantly, we identified that “Some people think I am less competent” (SIS2) was the most salient bridge symptom between perceived stigma and depressive symptoms in the network. Conceptually, SIS2 reflects social discrimination due to disease-related declines in capacity. Due in part to the rigidity of the dialysis treatment schedule and a decline in physical strength and energy, MHD patients are perceived as having limited competence and social value because they cannot participate in reciprocal exchanges. This can erode social connections and status, leading to a loss of social resources, and in turn potentially contributing to depression symptoms (Zhao et al., 2021). Additionally, this finding may be particularly relevant to Asian cultures, especially within Chinese society, where the concept of “face” is a deeply ingrained social phenomenon (Shen et al., 2022). In Chinese culture, personal value is often closely linked to recognition and respect from others. Therefore, the experience of being seen as “less competent” by others can lead to a perceived loss of face, making patients feel even more frustrated and worthless, thereby exacerbating their depressive symptoms. Of note is our finding that SIS11 “My job security has been affected” is not only a core symptom in the stigma network but also the second major bridge symptom in the combination network. The conclusion can likely be attributed to the following two factors.

Firstly, job insecurity, job loss, or unemployment caused by employment discrimination diminish the resources needed to cope with the perceived threat of stigma (Zhao et al., 2021; Pazderka et al., 2022). If the perceived threat of stigma surpasses an individual’s coping abilities, the learned helplessness associated with perceived stigma may contribute to the onset of depression (Xie et al., 2022). Additionally, individuals must confront anxieties related to potential losses of social standing, economic stability, and interpersonal relationships due to job insecurity. This can make them feel like a burden to society and their families, leading to feelings of worthlessness, guilt, and thwarted belongingness (Joiner, 2005), which can worsen depressive symptoms.

### Implications and recommendations

The greatest impact on the entire stigma model is expected from improvements in central symptoms (Castro et al., 2019). Therefore, intensive interventions by caregivers targeting central symptoms such as “Feel others avoid me because of my illness” (SIS5) and “Feel others think I am to blame” (SIS13) will be more effective in reducing overall levels of perceived stigma than addressing other symptoms.

To address the experience of perceived avoidance by others (SIS5), educational programs should be developed to enhance public awareness and understanding of hemodialysis, emphasizing that it is a life-saving treatment for patients with ESRD rather than a contagious or socially unacceptable condition. These programs can utilize various media platforms to disseminate accurate information, thereby reducing misconceptions and fears that may lead to avoidance behaviors. Secondly, support groups specifically tailored for hemodialysis patients can be established, providing a safe space for them to share their experiences. Peer support within these groups can foster a sense of belonging and validation, helping patients to better cope with the emotional challenges associated with their condition. Community integration initiatives can also be launched, such as organizing social events that involve hemodialysis patients in community activities, thereby reducing the perceived social distance and promoting a more inclusive society. Furthermore, Cognitive Behavioral Therapy (CBT) can employ cognitive restructuring techniques and behavioral validation exercises to help patients undergoing MHD correct their misinterpretations of others’ behaviors (such as misconstruing neutral actions as signs of rejection), thereby alleviating their perceived experiences of social avoidance.

“Feel others think I am to blame” (SIS13) originates from public misconceptions regarding the etiology of ESRD, representing a prototypical manifestation of internalized social stigma. A targeted intervention strategy should encompass educational initiatives aimed at both the public to dispel the notion that ESRD is a consequence of personal failing or moral deficiency. The internalization of external feedback from the public significantly perpetuates patients’ subjective perceptions of stigmatizing feelings (Yu et al., 2021). Therefore, interventions focused solely on reducing negative external feedback are inadequate. Studies have confirmed that cognitive interventions employing acceptance-focused strategies (Chambers et al., 2015), mindfulness-based interventions (Van der Gucht et al., 2018), and mindful self-compassion training (Eriksson et al., 2018) seem effective in further ameliorating the internalization of external discrimination. There is also a critical need for the effective implementation of these interventions.

Bridge symptoms are considered important treatment targets because the deactivation of these symptoms might prevent the development of comorbidity between mental disorders. “Some people think I am less competent” (SIS2) may be potential targets for further interventions aimed at improving both perceived stigma and depressive symptoms simultaneously in MHD patients. Previous discussions have highlighted that this bridge symptom are inextricably linked to the rigidity of the dialysis schedule and dialysis-related somatic symptoms. Enhancing the flexibility of the dialysis schedule and alleviating patients’ somatic symptoms should be a paramount objective for nursing staff. Therefore, it is important to encourage the early implementation of the Renal Rehabilitation Program (RRP) in clinical practice (Lin et al., 2021). This program should include multidisciplinary intervention programs such as medical care, education, counseling, diet management, and exercise training, aiming to help renal failure patients improve their functional status and career potential. Additionally, Rajkumar et al. (2022) integrated recommendations from dialysis patients and their caregivers across 86 countries to promote job security for dialysis patients. The most desired aspects were flexibility and control over work patterns, including decisions about dialysis methods and schedules. Home hemodialysis (HHD) and nocturnal hemodialysis (NHD) modalities have shown significant advantages due to their relatively flexible treatment plans, allowing patients more freedom in allocating time and not interfering with their normal daytime work (Tomori and Okada, 2018; Malavade et al., 2021). Successful HHD implementation requires guidance and support from medical teams, active participation from patients and families, and addressing challenges like lack of insurance coverage, high costs, and equipment complexity. Healthcare professionals must develop tailored training programs for home dialysis patients and caregivers and promote remote health monitoring systems to support government health initiatives.

Remarkably, “My job security has been affected” (SIS11) is not only a core symptom in the stigma network but also the second major bridge symptom in the combination network. Therefore, interventions targeting the decline in job stability hold significant potential for effectively reducing stigma and its comorbidity with depression among MHD patients. Mitigating the stigma associated with job instability (SIS11) requires comprehensive top-level design and policy support. These measures include encouraging businesses to create inclusive work environments and offer flexible work arrangements that cater to the specific needs of MHD patients. The government can encourage and support enterprises in offering appropriate incentives and assistance through policy frameworks and systems, ensuring that patients have access to employment opportunities during recruitment, training, and workplace adaptation. For the majority of dialysis patients who return to work, Erickson et al. (2018) suggest that adequate financial resources should be allocated for social support programs, such as psychiatric care, employment counseling, and vocational rehabilitation programs, to reduce the potential productivity loss of the dialysis population and further mitigate the economic burden of this disease on society and the barriers to returning to work.

### Limitations and future research

Several limitations should be considered in this study. Firstly, as the utilization of cross-sectional data limits the network to an undirected structure, drawing causal inferences within this framework becomes unattainable. A longitudinal investigation in the future research is imperative for assessing these associations. Secondly, the results should be interpreted with caution as the generated networks were based on group-level analysis. The following research can further investigate the individualized network in MHD patients, providing an opportunity to examine the dynamic fluctuations of perceived stigma and depression symptom. Thirdly, due to time constraints, the participants in this study were all from eastern China, with no samples from central and western regions. In the future, multicenter studies with larger samples could be carried out to improve the generalizability of the results. Finally, in our network study of perceived stigma and depression symptom, we just examined the items included in the scale we chose. Given the strong dependence of the results on various variables, despite the SIS and PHQ-9 has been widely used and validated among MHD patients, future research should also strive to conduct network analysis using more precise and comprehensive measurement tools in order to derive more accurate conclusions.

## Conclusion

This research reveals the core and bridge symptoms in different symptomatic (such as perceived stigma, depression symptoms, and the combination networks), which can be targeted for treatment personalization and aid in diminishing depressive symptoms and perceived stigma among MHD patients.

## Data Availability

The raw data supporting the conclusions of this article will be made available by the authors, without undue reservation.
